# Editorial: Flowering time control in agricultural and horticultural crops, volume II

**DOI:** 10.3389/fpls.2023.1183355

**Published:** 2023-06-14

**Authors:** Liang Wu, Leo F.M. Marcelis, Fanjiang Kong, Yang Zhu

**Affiliations:** ^1^ Hainan Yazhou Bay Seed Laboratory, Hainan Institute, Zhejiang University, Sanya, Hainan, China; ^2^ College of Agriculture and Biotechnology, Zhejiang University, Hangzhou, Zhejiang, China; ^3^ Horticulture and Product Physiology, Department of Plant Sciences, Wageningen University, Wageningen, Netherlands; ^4^ School of Life Sciences, Guangzhou University, Guangzhou, China

**Keywords:** flowering regulation, florigen, photoperiod, vernalization, plant architecture, nutrition

The control of flowering time is the key to reproductive success and strongly influences grain yield in crops. Mechanisms involved in flowering control are the determinants of plant breeding to produce novel varieties, which could better get adapted to local environments and changing climates. The key regulators of floral transition have been the subject of intense physiological studies for decades in model plant species revealing the genetic complexity of flowering time control. Flowering time appears to be well controlled by multiple pathways that are influenced by the environment in which the plant is grown and the developmental state of the plant. In recent years, by using wide varieties of genetic populations, a number of novel flowering-time-related genes or loci have been identified in all kinds of crop species playing regulatory roles in floral pathways *via* altering flowering behavior in crops. Due to topic diversity and many articles, this Research Topic is divided into two volumes that aim to illustrate recent discoveries in flowering time control in agricultural and horticultural plants. Particularly, volume II of this Research Topic is not just a collection of articles but includes innovative and interesting studies which will increase the visibility and transparency of flowering time control in plants.

A number of endogenous and exogenous factors regulate flowering time. Among other endogenous cues, FLOWERING LOCUS T (FT) is a mobile and florigenic protein that promotes the transition of flowering (Qin et al., 2019). To better understand the evolution of FT protein, Liu et al. performed a comprehensive phylogenetic analysis of FT genes in 47 sequenced flowering plants and the 1,000 Plant Transcriptomes (1KP) database focusing on monocots, especially cereals. According to their analysis, FT genes in monocots form three clades as a result of subsequent different selection pressures and amino acid substitutions, which eventually led to different expression patterns and functional diversification. Their study provides a global insight into FT genes’ evolution in monocots, investigating FT genes’ function in plant survival to adjust to the rapidly changing environment.

Similarly, Strejčková et al. collected 263 wild emmer wheat (WEW) genotypes for flowering time using genome-wide association mapping (GWAS), associating 16 SNPs with the heading date. As flowering time is controlled by photoperiod and vernalization, the authors sequenced the *VRN1* gene to discover new alleles and obtained full-length sequences of *VRN-A1* and *VRN-B1* genes in a panel of 95 wild emmer wheat, and uncovered a significant sequence variation. Phylogenetic analysis of *VRN-A1* and *VRN-B1* haplotypes revealed their evolutionary relationships and geographic distribution in the Fertile Crescent region, providing an available reservoir of allelic diversity that could be introduced into breeding programs of durum and bread wheat to expand the elite wheat gene pool.

Photoperiodism is known as one of the crucial biological modules for flowering control in plants (Johansson and Staiger, 2015). Unlike other short-day plants, soybean has a major maturity gene *E1* which plays a critical role in soybean photoperiodic responses. Therefore, Wan et al. employed CRISPR/Cas9-mediated targeted mutation of *E1* gene to investigate its function in photoperiod regulation and plant architecture. Four types of mutations in the *E1* coding region were generated with homozygous trans-clean mutants without T-DNA. The photoperiodic sensitivity of *e1* mutants decreased relatively to the WT plants and exhibited significant changes in the plant architecture. Further, to investigate *E1*-regulated genes related to plant architecture, the authors conducted RNA-seq to compare the gene expression profiles in the stem tip of the WT and the *e1* mutants. Their analysis indicated that key enzymes in GA synthesis and auxin efflux carrier proteins were upregulated in the *e1* mutant, suggesting that *E1* potentially regulates the branching type by modulating the genes involved in GA synthesis and auxin transport.

In another interesting study, Zhang et al. developed a novel experimental system characterized by photoperiodic responses for plant biological studies. They tested a distinctive artificially-made cotyledon-only plant (COP) using a photoperiod-sensitive soybean variety Zigongdongdou (ZGDD), and other photoperiod sensitivity varieties. ZGDD COPs showed a similar result to the integral ZGDD plants under natural day-length conditions. Moreover, at the molecular level, the key genes in the photoperiodic pathway, such as *E1*, *GmFT1a*, *GmFT2a*, and *GmFT5a* in the COPs, also showed the same photoperiod sensitivity as in the whole plants. In addition, a simpler material of COP with only one cotyledon and root was generated and found to be sensitive to photoperiod. Notably, the COPs are only one-fifth the height and one-third the maximum diameter of the intact plants grown in chambers 30 d after emergence. In conclusion, the authors suggested COPs as s potential and novel model material for studies of the developmental biology of soybean and other dicots in response to hormones, mineral elements, and pesticides.

Based on how plants regulate flowering time in response to different photoperiods, plants can be divided into three major types, namely short-day plants (SDPs), long-day plants (LDPs), and day-neutral plants (DNPs) (Li et al., 2022). The day length measurement system of SDPs is different from LDPs and controlled by a number of genes. In LDPs, such as Arabidopsis, CONSTANS (CO) integrates numerous internal and external signals for inducing photoperiodic flowering. At the same time, *OsHd1*, functional CO-like protein in rice, promotes and prevents flowering in rice under SD and LD, respectively. Subsequently, numerous dual-function regulators and phytochromes were also gradually identified (Li et al., 2022). In this regard, Sun et al. demonstrated the relationship among these modulators and proposed a regulatory network to increase our understanding of the critical day-length regulation mechanism and the negative response to photoperiod between SDPs and LDPs.

Following the discussion of photoperiod, Wang et al. comprehensively reviewed the molecular regulation of flowering and genotypic variations for molecular breeding and crop improvement. They discussed the regulation of flowering time in different crop species by focusing on how photoperiod-related genes facilitate adaptation to different local environments. They focused on the molecular networks regulating flowering time in different crops to maximize production and investigated variations in flowering time regulation with improved cultivars for future breeding programs.

Plants have developed highly systematic, complex, and specialized nutrient sensing and signaling systems to respond to varying nutrients availability in the soil (Podar and Maathuis, 2022). Nutrition affects plant growth and development, including flowering, where plants require the balanced consumption of nutrients. Some nutrients act as signals and affect flowering regulation, which is intimately associated with nutrient use efficiency (NUE) and crop yield (Jia et al., 2021). Here, Zhang et al. reviewed the current knowledge of the relationships between macronutrients (primarily nitrogen, phosphorus, and potassium) and flowering to deepen our understanding on how plant nutrition affects flowering. The authors suggested improvement in NUE by coordinating flowering time in an effective way to increase crop yield.

Altogether, these recent studies collectively offer a thorough overview of the fundamental mechanisms of flowering time control and related genes in agricultural crops. This study subject highlights cutting-edge and emergent topics in the field and opens up new area of research that will motivate scholars with diverse research interests.

## Author contributions

All authors listed have made a substantial, direct, and intellectual contribution to the work and approved it for publication.
